# News and Notes

**Published:** 1890-02-08

**Authors:** 


### Notes and News.

Collected and Communicated.

The Master of Lewisham Workhouse is being called upon to account for certain toys and sixpences sent by *Truth* for the children there and never distributed. The Master bears a good character, and seems to have erred through error of judgment. Some of the children he considered too young to have sixpences, and the toys he considered too numerous to be all given at once. The difference between *meum* and *tuum* is hard to impress on certain minds, the general belief being that in public institutions the inmates have no right to possess aught of their own. But you have no more right to keep back a present sent a pauper than a present sent a prince. It must be evident to all readers of the daily papers that masters of workhouses are either a much-maligned race, or else an exceptionally arrogant and unkind group of men.

Returning to the subject of hospital balls, and especially to the dance at the Homerton Asylums Board Hospital, we emphatically state that for the nurses to dance in the children's ward with acute cases there, and for nurses to attend from the diphtheria and enteric wards, was a great mistake. We believe the nurses are permitted to dance in their own sitting-room every evening from eight to ten, and with this wise, yet generous permission, they should have been content. The matron at Homerton has resigned—very naturally so; there was no other course open to her when her authority was put aside. We can only hope the Asylums Board will make a thorough inquiry into the matter, or they will have considerable difficulty in securing efficient matrons in future. The Nottingham General Hospital held its annual ball on January 22nd, in the old wing, which was prettily decorated for the occasion. Dancing commenced at nine p.m., and was kept up with great spirit till a late hour. Amongst the guests were several members of the present and past medical staff, and many old friendships were renewed in the pauses of the dancing. Everything is wisely arranged at the Nottingham Hospital, so that the patients are not neglected, and the nurses gain a well-earned pleasure.

The Samaritan Fund in connexion with the Durham County Hospital issues its annual report. During last year 15 patients were sent to convalescent homes, and several were supplied with surgical appliances, or otherwise aided. The nurses visited 411 patients, and these, when destitute, were supplied with milk, meat, and wine tickets. The society is managed by ladies, Mrs. Lake being president.

Moreton-in-Marsh Cottage Hospital treated 104 cases last year, the total average cost per case being £3 5s. 7d. Mr. Style is proposed as assistant surgeon in place of Mr. Hurlbutt resigned, but Mr. Style will not accept the post unless he has also a seat on the committee. The matron, Miss Horne, was warmly thanked for her devotion to her patients, and her strict economy in household management.

The Nubian Minstrels, on January 24th, gave at the Parochial Hall, Balham, a voluntary entertainment in aid of the funds of the Royal Hospital for Children and Women, Waterloo Bridge Road, S.E. Their clever performance was much appreciated by the matron and nurses of the hospital who were present, as well as the audience interested in the charity, and was in all ways a success.

An amusing instance of red-tapeism is reported from America. A county hospital has been built in Omaha, and the contractors can get no one to take it off their hands. The county commissioners will only accept it through the architect, and the architect has vanished, no one knows where. It seems not improbable that the building supposed to have been erected for the benefit of mankind, will be allowed to fall into decay for the lack of a little common sense.

The Rev. W. T. McCormick has written to the local press on the subject of the non-contagious nature of leprosy. Mr. McCormick has visited the leper asylums of Norway and Africa, and believes the disease to be purely hereditary. Unluckily, we cannot accept this view without further evidence. How is it that our sailors and soldiers develop leprosy in India if the disease is non-contagious? It almost seems as though Mr. McCormick does not distinguish the difference between contagion and infection.

Leeds Hospital for Women issues its annual report. The Hospital records for 1889, being the thirty-seventh year of its existence, show an increase of in-patients, and a slight decrease of out-patients. During the past year 1,212 patients have been treated. Of these 211 were in-patients, being an increase of 27 over the number in 1888. The out-patients have been medically attended 3,839 times, against 4,154 in the previous year. Although it is hardly possible, in a small special hospital, to draw any serious conclusions from comparative statistics as to patients—a very limited number materially increasing or diminishing the appearance of work done—yet it is satisfactory to know that the time each patient has on the average occupied a bed in the Hospital has been reduced from thirty-six to thirty days. The committee invited applications from duly qualified surgeons to fill the vacancy caused by Mr. Scattergood's retirement. There were five candidates, and the voting resulted in the election of Dr. J. B. Hellier, whose qualifications and experience warrant the belief that he will prove to be a valuable officer of the hospital. In the room of Dr. Clifford Allbutt, the committee elected Dr. J. E. Eddison, senior physician to the Leeds Infirmary, who has accepted the position of honorary consulting physician.

The following vacancies are announced this week, the amount of the salary in each case being given after the name of the institution:

**Resident Medical Officers.**

City of London Hospital, Victoria Park. House Surgeon—Board, &c.
Great Northern Hospital. House Surgeon—£60, board, &c.
Royal London Ophthalmic Hospital. Junior House Surgeon.
St. Mark's Hospital. House Surgeon—£50, board, &c.
Salford Union. Assistant Medical Officer—£140, rooms, &c.

**Non-Resident Medical Officers.**

Gourock District. Medical Officer—£25.
Plymouth Public Dispensary. Second House Physician.
West End Hospital. Dental Surgeon.
London School Board. Medical Officer—£400.

The death of Sir William Gull, which occurred last week, removes one of the most celebrated physicians from our midst. Sir William was 73 years of age, and had been in ill-health for several years. The funeral took place on Monday at Thorpe-le-Soken; Major-General Sir C. Teesdale was present as the Prince of Wales's representative. Over 100 beautiful wreaths were sent.

The first festival dinner of the East-end Mothers' Home (396, Commercial Road, E.), formerly the Shadwell Lying-in, took place at the Whitehall Rooms, Hôtel Métropole, on Wednesday (29th ult.), the Right Hon. the Earl of Aberdeen in the chair. This maternity hospital at Shadwell has now been established some five years, during which time nearly five hundred women have been treated, with what success is shown by Dr. Playfair's statement at the annual meeting last year, that while the percentage of deaths in child-birth was one in 128, the percentage of deaths in the Mothers' Home was *nil*. There certainly had been one death at the institution, but this was the result of starvation before the patient was admitted. As an institution it stands alone in the large and crowded region of East London, and its object is one of the most worthy ones one can very well imagine. It seeks to bring to the poor in this part of the metropolis, as far as its accommodation will allow, the quiet and privacy, good attendance, and skilled nursing which is the *sine quâ non* of rich people under circumstances which generally cause much extra expense, anxious care, privation, discomfort, illness, and which result sometimes even in death. The excellent results which up to the present have accrued from its work are due no doubt in a very large measure to the untiring energy and remarkable administrative skill of the lady superintendent, Mrs. Ashton Warner, who was practically its founder, and who has devoted herself unsparingly to the proper care and comfort of those who may come under her charge.

In proposing the toast of the evening the chairman referred to the last financial statement of the institution, which, he said, showed a remarkably small proportion of office expenses as compared with the total expenditure. The latter amounted to £1,383 3s. 11d., while the salaries and wages of nurses and servants only came to £206 13s. 8d. As to the apparently large expenditure, his lordship said that though it might seem large for the number of patients treated, yet if the Home is to be conducted so as to carry out the objects in view, there must be a larger expenditure as compared with that of other institutions. Then, too, it would be possible to treat a very much larger number of patients for a very slight increase in the expenses, and, therefore, with an extension of the accommodation they might expect a very large increase in the work done by the hospital. The Lady Superintendent during the course of the evening announced that £675 8s. had been contributed that day towards the Mothers' Home. Miss Marguerite Hall and Mr. Oswald agreeably varied the usual programme by their excellent renderings of songs and duets, Miss Mary Carmichael acting as accompanist.

Another onslaught on hospitals appears in the *Liverpool Post* in answer to Mr. Armstrong's letter which we quoted last week. The writer says: "Our hospitals are floated to treat diseases which originate through carelessness or neglect. Who could truthfully say that hospitals for illegitimate children, for cancer, for epilepsy, for diseases due to drink and prostitution are Christian institutions? Are not all our deformities and inmates of homes for idiots and imbeciles a standing record against us; and a plain proof that nature will resent downright carelessness and ill-usage? Every deformity, and every departure from the normal state, points to the hard fact that a law of health has been broken. But so long as men and women know that they can contract disease and hand it on, or receive treatment at a charity, little good will be effected. Disease and suffering we must have, but, like every other evil, we would be content to accept as small a grant as possible. If our hospital authorities would follow up and trace to their origin the different diseases, then we might hope for some mitigation of the evils." It does not seem to occur to these correspondents that it is the duty of sanitary inspectors, householders, and others, to secure healthy conditions which shall partly do away with disease. We should not wonder if both these writers owned property not properly drained, and not made as healthy as it might be. The work of prevention is chiefly in other hands, but the disease once there the work of cure is with the hospitals. It is useless to complain of the hospitals, but be sure the authorities of these institutions will be glad if you will help to empty their wards by increasing the average health of the nation.

The annual general meeting of the governors of the Metropolitan Hospital was held on Monday in Kingsland Road, Mr. Joseph Fry, Chairman of the Committee of Management, presiding. Mr. C. H. Byers (the Secretary) read the annual report, in which the committee congratulated the governors upon the increased usefulness of the Hospital. The number of in-patients treated had been 489, as against 247 in 1888, and the out-patients had numbered 9,482, against 4,100 the previous year. The committee were glad to be able to report the continued success of the provident department. During the past year 3,681 membership books had been issued, making the total number of membership books at the end of 1889—8,126, representing over 16,000 lives. The attendances of provident members had numbered 42,718, against 29,088 in 1888. Those facts and figures proved that the poor really appreciated the provident principle, and confirmed the committee in their opinion that in starting and carrying on the scheme they had met an actual want, and had not pauperised the poor in doing so. The total receipts for the year had amounted to £4,709 12s. 8d., and the expenditure to £5,818 0s. 1d. Among the donations received last year was one of £1,000 from the Earl of Wilton, and as a slight recognition of that gift the committee had named a bed in the men's ward the "Wilton." Under the scheme for the disposition of the £10,000 left by Mr. J. Jackson to build a cottage hospital and a soup kitchen for the poor of Shoreditch, but whose wishes could not be literally carried out, the Hospital had received £2,614 17s. 9d. towards the building fund, and a further amount of £2,941 London and North-Western Railway Guaranteed Stock, and £2,964 North-Eastern Four per Cent. Preferential Stock was held in trust for the Hospital by the Commissioners for Charitable Trusts, who would pay the interest from those stocks at intervals to the account of the Institution. In consideration of that large sum the committee had set aside four beds for men, four for women, and four for children, naming them "Jackson" beds, with the proviso that they are for the benefit of poor patients from the parish of Shoreditch. Although the number of beds had been increased from 51 to 76, there was still room for an additional 80 or 90.

